# Blood stasis in the descending thoracic aorta and its relationship to cardiac functional parameters

**DOI:** 10.1186/1532-429X-13-S1-P172

**Published:** 2011-02-02

**Authors:** Lauren A Simprini, Gaby Weissman, Anthony R Fuisz

**Affiliations:** 1 Washington Hospital Center, Washington, DC, USA

## Background

Spin echo Axial T1 images are used to evaluate the great vessels and cardiac structures. In some patients with depressed left ventricular ejection fraction (LVEF) the sequence fails to null blood of the descending aorta (dAo) at the level of the main pulmonary artery (MPA). This may represent stasis of blood from the aorta and reflect a decreased LVEF, stroke volume (LVSV) and/or cardiac output (CO). This finding has not systematically been studied. We evaluated the relationship between opacification of the descending aorta and cardiac functional parameters.

## Methods

148 consecutive cardiac MR scans performed between 7/2010 and 9/2010 that included evaluation of left ventricular function were reviewed. The T1 axial images (SE-EPI TR750TE20Flip90) were evaluated for opacification of some or all of the dAo at the level of the MPA. This was also identified in either the slice above or below this level to be considered a positive finding. The values of LVEF, SV, and CO as determined by manual tracings and application of Simpson’s rule were obtained (ViewForum, Philips, Best, The Netherlands).

## Results

Mean LVEF value for patients with no opacification was 60% while those with any amount of opacification had a mean LVEF of 36% (p < .0001). Specificity and sensitivity for LVEF < 55 was 97% and 32% respectively, for LVEF < 45 was 95% and 40% respectively, and for LVEF < 35 was 93% and 53% respectively. The mean SV value for patients with no opacification was 96 ml while those with any amount of opacification had a mean LVSV of 58 ml (p < .0001). Specificity and sensitivity for LVSV < 50 ml was 92% and 53% respectively. The mean CO value for patients with no opacification was 6.2 L/min while those with any amount of opacification had a mean CO of 4.5 L/min (p < .0001). Specificity and sensitivity for CO < 5.0 L/min was 96% and 38% respectively.

## Conclusions

Opacification of the descending aorta at the level of the MPA is a specific but not sensitive finding for depressed LVEF and lower than normal LVSV and CO. When this finding is observed, further evaluation of a patient’s ejection fraction should be considered.

**Table 1:** Specificity and sensitivity of descending aorta opacification

	Specificity	Sensitivity
LVEF < 55	97%	32%
LVEF < 45	95%	40%
LVEF < 35	93%	53%
LVSV < 50 ml	92%	53%
CO < 5 L/min	96%	38%

